# Silk Fibroin as a Functional Biomaterial for Tissue Engineering

**DOI:** 10.3390/ijms22031499

**Published:** 2021-02-02

**Authors:** Weizhen Sun, David Alexander Gregory, Mhd Anas Tomeh, Xiubo Zhao

**Affiliations:** 1 Department of Chemical and Biological Engineering, University of Sheffield, Sheffield S1 3JD, UK; WSun10@sheffield.ac.uk (W.S.); d.a.gregory@sheffield.ac.uk (D.A.G.); matomeh1@sheffield.ac.uk (M.A.T.); 2 Department of Material Science and Engineering, University of Sheffield, Sheffield S3 7HQ, UK; 3 School of Pharmacy, Changzhou University, Changzhou 213164, China

**Keywords:** silk fibroin, biomaterial, scaffold, tissue engineering

## Abstract

Tissue engineering (TE) is the approach to combine cells with scaffold materials and appropriate growth factors to regenerate or replace damaged or degenerated tissue or organs. The scaffold material as a template for tissue formation plays the most important role in TE. Among scaffold materials, silk fibroin (SF), a natural protein with outstanding mechanical properties, biodegradability, biocompatibility, and bioresorbability has attracted significant attention for TE applications. SF is commonly dissolved into an aqueous solution and can be easily reconstructed into different material formats, including films, mats, hydrogels, and sponges via various fabrication techniques. These include spin coating, electrospinning, freeze drying, physical, and chemical crosslinking techniques. Furthermore, to facilitate fabrication of more complex SF-based scaffolds with high precision techniques including micro-patterning and bio-printing have recently been explored. This review introduces the physicochemical and mechanical properties of SF and looks into a range of SF-based scaffolds that have been recently developed. The typical TE applications of SF-based scaffolds including bone, cartilage, ligament, tendon, skin, wound healing, and tympanic membrane, will be highlighted and discussed, followed by future prospects and challenges needing to be addressed.

## 1. Introduction

Damaged and degenerated tissue, as well as failed organs, are some of the most serious issues in human healthcare, generating many challenges in modern medicine. For example, musculoskeletal tissue (bone, tendons, and cartilage), as well as the peripheral nervous system, are easily impaired by trauma and degenerative diseases such as osteoarthritis. This affects millions of people worldwide, severely affecting the quality of life and resulting in extreme pressure on healthcare systems worldwide [1,2]. Typically, autografts and allografts are the common clinical techniques to replace damaged tissues, but restricted by various factors, such as lack of tissue that can be removed from the patient in healthy areas, as well as a shortage of suitable donors [3]. Success rates of allografts can be low as tissue from others may have an immune response. In the case of extensive damage, large surface areas of defects, it is hard to source suitable material in time leading to low success rates [3,4,5]. It is for these reasons that tissue engineering (TE) has attracted increasing attention as the alternative method to produce patient-specific tissues for repair and replacement applications.

TE combines several principles and methods to regenerate damaged tissues or organs by restoring, maintaining or improving tissue functions. Furthermore, TE relies extensively on the use of biocompatible scaffolds which are typically seeded with cells and contains supportive moieties such as growth factors [6,7]. Regardless of the tissue types, there are several key factors that should be considered when designing a scaffold. These include biocompatibility, biodegradability, mechanical properties, structure, and fabrication methods [7,8]. The extracellular matrix (ECM) secreted from tissues or organs is an excellent natural option as a scaffold material for TE, and exists in a state of “dynamic reciprocity” with resident cells [9]. Therefore, ECM components such as collagen [10], fibronectin [11], laminin [12], elastin [13], and glycosaminoglycan [14] have been widely used as natural scaffold materials to support tissue regeneration applications. In addition, other natural polymers such as alginate, [15] cellulose [16], and chitosan [17] have also been used in TE. Although the natural polymers discussed above have demonstrated promising results, these materials also have many drawbacks including high cost, poor mechanical properties, and large batch to batch variation, making them difficult to be applied to clinical applications [18]. On the other hand, synthesized polymers, such as polylactic acid (PLA), polyurethane (PU), poly(lactide-co-glycolide) (PLGA), and polycaprolactones (PCL), have been widely used in TE due to their good mechanical properties and degradation rates [19]. However, many degradation products of these polymers comprise of acidic compounds that are harmful to the body and can cause undesired immune responses. As most of the natural and synthesized polymeric scaffolds possess their inherent limitations, finding a biomaterial that combines the goodness of both natural and synthesized polymeric materials have become the aspirations of researchers in the last decades [20]. Recent studies have explored the possibilities of silkworm silk as an excellent biomaterial for TE scaffolds.

Silkworm silk has been commercialized in the traditional textile industry for more than 4000 years, due to its outstanding physical properties, such as lustre, lightweight, flexibility, and strong mechanical strength [21]. Moreover, silk has been approved by the Food and Drug Administration (FDA) for use in sutures and has been applied to biomedical applications for the last 2 decades [22,23]. Silk fibroin (SF), extracted from silkworm silk, is a unique natural protein that has been used as a potential biopolymer for TE, due to many desired physiochemical properties such as excellent biocompatibility, biodegradability, bioresorbability, low immunogenicity, and tuneable mechanical properties [24,25,26,27]. SF also can be combined synergistically with other polymers to form SF-based composite scaffolds, that can further promote cellular behaviour (e.g., differentiation, proliferation, and attachment) [28,29,30]. Further to this it is possible to fabricate SF-based biomaterials into various material formats, such as films [31], hydrogels [32], sponges [33], 3D structures [34], and nanoparticles [35]. In this review, we introduce the sources, material properties, fabrication techniques and applications of silk scaffolds with an emphasis on bone, cartilage, ligament, tendon, skin and wound tissue regeneration.

## 2. Sources of Silk and Silk Fibroin

Silks are proteins which are produced within glands after biosynthesis in epithelial cells. There are over 200,000 different silk-producing arthropods that exist in nature [35]. Out of these, there are many different taxonomic silk-producing families such as silkworms, spiders, lacewing, glowworm, and mites, some of which can spin silk into fibers during their metamorphosis (cocoon generation) [36,37]. Recently, Yoshioka et al. [38] discovered that the Psychidae family, also known as bagworm moths, are thought to produce the toughest form of moth silk currently known. Silks originating from silkworms and spiders are the most commonly used for biological applications [39,40,41]. However, in the case of spider silk once it is spun and contacts air it hardens, which restricts mass production of spider silks. Compared to spiders, the yield of fibers obtained from one silkworm cocoon is around 10 fold that of the ampullate gland of a spider [36,42]. Although researchers have used a biomimetic spinning process to replicate spider silks, producing spider silk-like fibers with mechanical properties similar to natural spider silk fibers is challenging [43]. Andersson et al. [44] designed a chimeric recombinant spider silk protein that can produce large quantities artificial spider silks via a bacterial shake-flask culture. The mechanical properties of these artificial spider silks are highly reproducible. However, the reported ultimate tensile strength and toughness are still lower than the of native spider silk fibers.

Bombycidae and Saturniidae are known to play the most important roles in silkworm silk research, which feed on either the mulberry tree (Bombycidae) or other food sources, the latter being regarded as non-mulberry (Saturniidae) silks. The most common silk originates from *Bombyx mori* (*B. mori*), a mulberry feeding silkworm that produces higher quality fibers than most Saturniidae [45,46]. Additionally, unlike other silk moths, over the last 5000 years, B. mori was domesticated from an ancestral species in China and has since then been extensively reared worldwide to obtain its silk [47]. B. mori silkworm cocoons consist of 75–83.3% SF and 16.7–25% of sericin [48]. SF is a semi-crystalline structured protein, functioning mainly for its load-bearing capacity. Sericin on the other hand is an amorphous protein-polymer functioning as a gumming agent [49]. It has been found that sericin-free fibroin fibers show better mechanical properties than sericin encased fibroin, where a 50% increase in tensile strength, a modulus of up to 15–17 GPa and strain at breakage reaching 19% has been observed [50]. Furthermore, sericin-free fibroin fibers also show better biocompatibility in-vitro and in-vivo according to previous reports [51]. In addition, sericin has been shown to cause inflammation [52]. Therefore, sericin proteins are often removed from SF to ensure biocompatibility in TE applications.

Sericin is removed from the SF fibers by a degumming process, which is normally carried out under boiling alkaline conditions [53]. Researchers continuously work on improving the degumming process which typically requires reagents and organic solvents to obtain higher quality of pure SF. The sodium carbonate (Na_2_CO_3_) degumming method has, at present replaced the standard Marseilles soap method, and is now the most used method due to being rapid (~30 min) and low cost [54,55]. It is worth noting that, after degumming, the average diameter of silks fibers was reduced to 10 to 25 μm [56].

## 3. Properties of Silk Fibroin

### 3.1. Structure of SF

**Figure 1:**
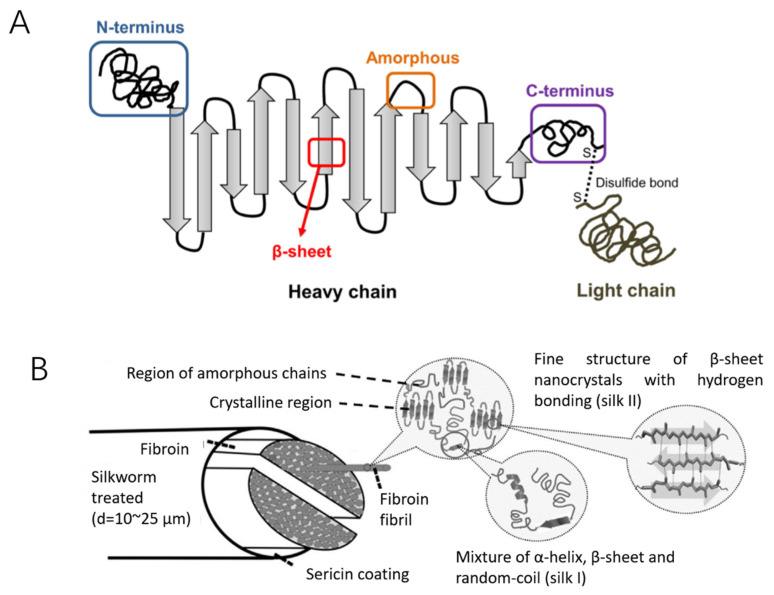
Schematic diagram of the silk structure. (**A**) heavy chain (i.e., N-terminus, β-sheets, Amorphous and C-terminus) and light chain which linked via disulphide bonds. Reproduced with permission from [40] (**B**) silkworm thread, fibril overall structure and silk fibroin polypeptide chains. reproduced with permission from [39].

SF consists of two main chains, a heavy (H-) chain (390 kDa) and a light (L-) chain (26 KDa), which are linked via disulphide bonds to form a H-L complex (Figure 1A) [39,40,57,58]. P25 (25 KDa) is a glycoprotein includes Asn-linked oligosaccharide chains, which is hydrophobically linked to H-L complex [59]. The H-chain, L-chain, and P25 are the three polypeptides that form the cocoon of B. mori and are found at a molar ratio of 6:6:1, respectively [60]. The amino acid sequence of the H-chain consists of Glycine (45.9%), Alanine (30.3%), Serine (5.3%), Valine (1.8%), as well as 4.5% of 15 other amino acid types. The Gly-X (GX) dipeptide motif repeats account for 60–75% of the H-chain. The hydrophobic residues of the dipeptide repeats can form stable antiparallel β-sheet crystallites. The two hexapeptides occupy 70% of the GX dipeptide motif region, for which the peptide sequences are known to be Gly-Ala-Gly-Ala-Gly-Ser and Gly-Ala-Gly-Ala-Gly-Tyr [61,62,63,64]. Silk I and silk II are the dominant crystalline structures of SF (Figure 1B), where silk I is a metastable crystalline structure that includes bound water molecules and silk II is the most stable state due to strong hydrogen bonding between adjacent peptide blocks, resulting in increased mechanical properties including rigidity and tensile strength [39,65,66].

The secondary structure obtained from regenerated silk fibroin (RSF) solutions contains crystalline and amorphous structures, which will be discussed below. In a crystalline structure, silk includes β-turns (silk I) and insoluble structures formed by folded β-sheets (silk II), while in an amorphous state silk consists of α-helices, turns and random coil structures [67]. Methanol or potassium chloride can easily convert silk I to silk II, a process which is widely used for biomaterial engineering applications [34]. Silk III is the unstable crystal structure of SF, which exists at the air–water interface of RSF solutions [68].

### 3.2. Mechanical Properties

SF fibers have demonstrated outstanding mechanical properties [56,69,70]. These include a large break strain (4–26%), ultimate strength (300–740 MPa) and toughness (70–78 MJ m^−3^) [70]. In addition, the reported toughness of SF fibers is higher than many synthetic fibers such as Kevlar (50 MJ m^−3^), carbon fiber (25 MJ m^−3^), and some collagens such as tendon collagen (7.5 MJ m^−3^) [43,71]. In addition, SF fibers exhibit the highest strength among common natural materials such as wool, resilin, elastin, byssus, and cotton, as well as some synthetic fibers such as synthetic rubber and viscose rayon [43]. Considering these strong mechanical properties of SF, many researchers have used SF as a scaffold material for load-bearing TE applications, especially in musculoskeletal TE [1]. It is, however, important to note that SF scaffolds in biomaterial engineering are normally made from RSF solutions and the produced scaffolds are brittle and weak. This is because RSF lacks hierarchical and secondary structures compared to unprocessed raw SF fibers [72]. In order to ensure RSF has good mechanical properties, many different strategies have been trialed. For example, the breaking stress of RSF fibers, made via a dry-spinning technique, was 252 MPa, 28.6% less than raw SF fibers (353 MPa), whereas, the breaking stress of RSF and graphene oxide composite silk fibers (dry-spun from a mixed dope of RSF and graphene oxide at mass ratio 1000/1) was 435 MPa [24]. Amongst others crosslinking, [32] porogens [73] and 3D bioprinting [34] technologies can be used to improve mechanical properties of RSF produced silk scaffolds. The resulting SF-based scaffolds are therefore sufficiently strong to allow handling during surgical procedures needed for implantation and have mechanical properties closely resembling the native tissue being repaired thus allowing for optimal repair conditions of the area in question.

### 3.3. Biocompatibility

Biocompatibility is a key factor for the implementation of successful scaffolds, which enables cells to adhere to scaffold surfaces and migrate into the scaffold undergoing proliferation and differentiation within the scaffold. In addition, it is important for the scaffold to cause no or a negligible immune reaction after implantation [6]. SF is known to be a biologically inert and therefore biocompatible natural polymer [26]. Since 1989, SF has been shown to have blood compatibility in in-vivo experiments [74]. In 1993, SF was approved by the FDA as a biomaterial for use in as a suture material [26]. In 1995, Minoura et al. [75] conducted pioneering research and successfully grew fibroblast cells on SF coated films. SF has more recently been used as an alternative to collagen in cell culture to guide bone regeneration in rat calvarial defects, for example, demonstrating that SF membranes can replace the collagen membranes [76]. In vitro studies showed that there is no significant macrophage response to SF films [77] or fibers [23]. In addition, the in vivo inflammatory reaction to SF films is similar to that of collagen [78].

### 3.4. Biodegradability and Bioresorbability

**Figure 2:**
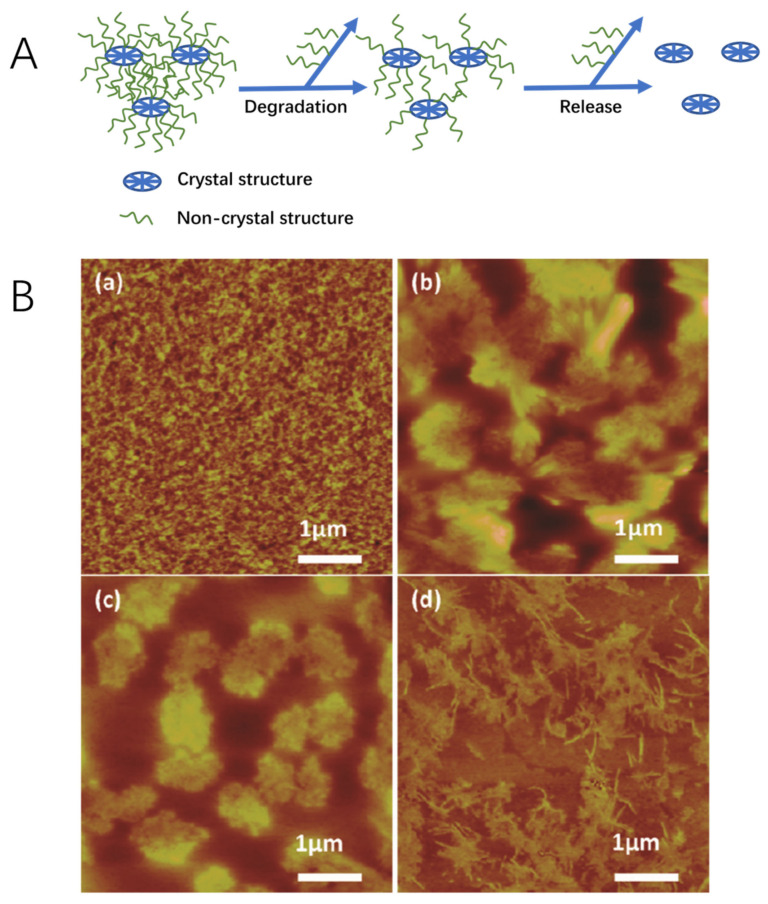
(**A**) Schematic illustrating the SF degradation process mechanism. (**B**) Representative AFM images of (**a**) pure protease XIV solution and differently fabricated SF films: (**b**) slow drying process, (**c**) water annealing treatment, and (**d**) stretching treatment, after 12 h of exposure to protease XIV solution. The degraded SF particles that dissolved in protease XIV can be seen in (**b**), (**c**), and (**d**). Reproduced with permission from [82].

Biodegradability and bioresorbability are important features to successful scaffold materials, as the scaffolds should gradually be replaced with the patients’ own cells and ECM over the course of recovery [79]. Therefore, it is important that by-products of biodegradation are non-toxic and do not interfere with other tissue, organs, and functions when being metabolized in the body. SF is an enzymatically degradable polymer and has been shown not to cause an immunogenic response [80]. The degradation process starts when enzymes are adsorbed onto the surface of the SF scaffold via surface-bonding domains. The enzymes then digest SF via hydrolysis of ester bonds [19,80,81]. The mechanism of SF degradation is shown in Figure 2A [82]. Non-crystalline SF structures (hydrophilic blocks) were degraded in an enzyme solution resulting in hydrophobic crystal structures and then further dissolved in enzyme solutions. SF can be proteolytic degraded through enzymes, such as α-chymotrypsin, protease XIV and collagenase IA [80,83,84]. Protease XIV, obtained from *Streptomyces griseus*, has shown a higher SF degradation activity in comparison to α-chymotrypsin and collagenase IA. This therefore meant that protease XIV degraded SF achieved the lowest average molecular weight of SF residues [83]. It is for this reason that protease XIV is the most commonly used enzyme for silk degradation. The preparation methods of SF also affect the degradation process, which can lead to different morphology of SF particles that dissolved in enzymes (Figure 2B) [82]. As the degradation products of SF are amino acids and peptides, they are easily absorbed in-vivo [80]. In vivo studies undertaken on SF porous scaffolds implanted in Lewis rats showed that the scaffolds decomposed within 8 weeks. After 1 year, the implanted scaffolds were fully degraded, due to macrophage degradation [85]. This proves that SF scaffold are not only biodegradable, but also is bioresorbable.

The degradation of native silk fibers is much slower compared to that of RSF silk scaffolds. This is due to the fact that native silk fibers have a higher content of β-sheet secondary structure than RSF structures have [86]. The degradation rate of SF is therefore highly dependent on the amount of β-sheet secondary structures present. For example, RSF films obtained by methanol treatment, converting water-soluble silk I to water insoluble silk II structures, resulted in a higher amount of β-sheet structures [87] in contrast, RSF films obtained via a slow air-drying process possess a lower content of β-sheet structures [88]. The latter therefore resulted in faster degradation rates. γ-radiation also has been shown to promote SF fiber degradation, due to the conversion of silk II to silk I [89].

## 4. Silk Fibroin Dissolution Techniques

**Figure 3:**
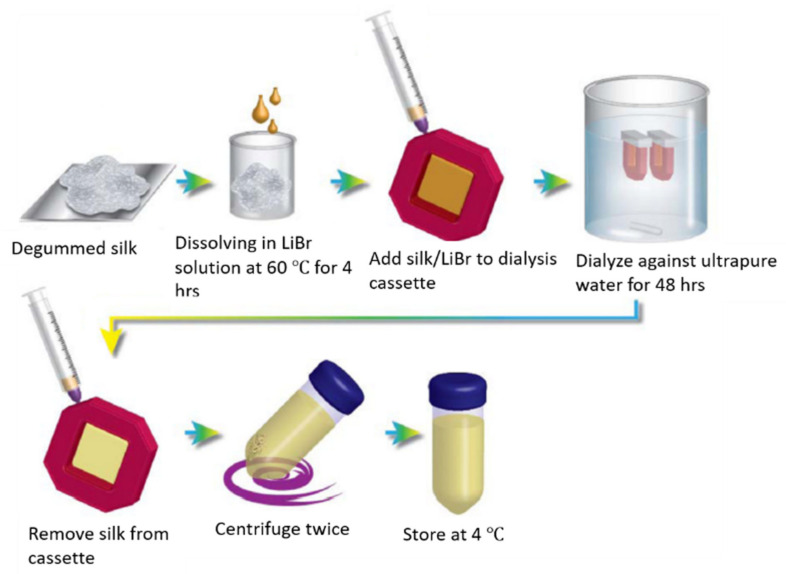
A schematical representation of the LiBr dissolution process to obtain RSF solution. The degummed silk is dissolved in 9.3 M LiBr solution at 60 °C for 4 h. The obtained solutions are dialyzed against ultrapure water to remove salt. Until a conductivity of < 5 µS is reached, RSF solutions are centrifuged twice and stored at 4 °C. Reprinted with permission from [21].

Proper dissolution of SF is an essential step before processing SF into different structures for various TE applications [90]. Therefore, a robust protocol for the complete and correct dissolution of silk cocoons to produce RSF is required. SF is insoluble in organic solvents and water, because of its tightly packed structure which has a high content of β-sheet structures [87,91]. To obtain an aqueous SF solution, it must undergo a water-based dissolution process [92]. As RSF solutions are used for biological applications, strong and toxic solvents and solutions should be avoided during the dissolution process. Typically, concentrated salt solutions with various concentrations of salt ions (Ca^2+^; Sr^2+^; Li^2+^; Zn^2+^) [93] in combination with anions (Cl^−^; Br^−^; SCN^−^) [94] were employed to dissolve SF fibers. These include the very well-known 9.3 M lithium bromide (LiBr) solution method (Figure 3), [21,92] as well as 9 M lithium thiocyanates (LiSCN) methods [95]. Another common method uses Ajisawa reagent [93], which consists of a ternary (CaCl_2_/EtOH/water) solvent (1:2:8 molar ratio) solution to dissolve SF. However, all these aqueous methods require a final dialysis step against pure DI^−^ water or appropriate buffers to remove salt ions from the RSF solutions. Recently, Ajisawa’s reagent has increasingly been applied in SF dissolution, due to its cost efficacy. However, compared to the LiBr method, Ajisawa’s reagent appears to lead to a complete unfolding of the silk polymers, which are therefore more prone to form β-sheet structures and aggregate during dialysis [96]. Zheng et al. [96] adapted this method dissolving degummed silk fibers in Ajisawa’s reagent at 80 °C for 2 h and then dialyzing against urea solution with a stepwise decrease in concentration. When the SF solution was dialyzed against water and urea (4 M constant concentration) solutions for 30 h (referred to as Silk-TS-0), the hydrodynamic radius of RSF ranged from 100 to 1000 nm. However, when the SF solution was dialyzed against 4 M urea for 3 h, then in 2 M urea for 3 h, followed by 1 M urea for 3 h and then water for 30 h (referred to as Silk-TS-4210), the hydrodynamic radius range of RSF solution reduced to 5–11 nm. In addition, Silk-TS-4210 had small aggregates (<10 nm), and a low content of β-sheets (≈15%) compared to Silk-TS-0, an outcome similar to RSF via the LiBr method [96].

## 5. Morphological Diversity of Silk Fibroin Scaffolds

**Figure 4:**
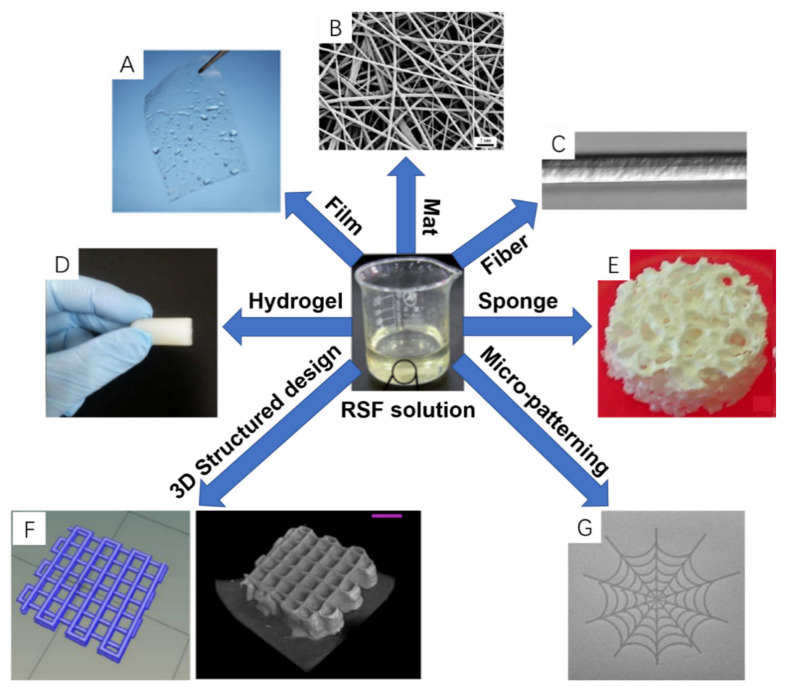
SF-based scaffolds with different representative structures: (**A**) Film; (**B**) Mat; (**C**) artificial fiber; (**D**) Hydrogel; (**E**) Sponge; (**F**) 3D structure design and printed scaffold; (**G**) Inkjet-printed silk pattern. Reprinted with permission from [33,97,98,99,100,101,102].

RSF solutions have been used to fabricate SF-based scaffolds with different structures (Figure 4), including films, mats, artificial fibers, hydrogels [97,98,99,100], and sponges [33]. Different techniques used for micro-patterning and 3D structures fabrication are described in the following sections [101,102].

### 5.1. Films

Spin coating and vertical deposition are the main techniques used to fabricate RSF films. In the case of spin coating, RSF solution and ethanol are alternately coated onto substrates. As previously described ethanol is able to convert the structure of RSF from high content α helices (Silk I) into beta sheet conformation (Silk II). The ethanol concentration used can affect the surface properties of RSF film. If the concentration of ethanol is less than 80%, the outermost surface of the treated film will have a hydrogel structure [103]. However, when using 90% *w/v* ethanol, the silk films surface become rigid, and cells show better adhesion [103]. Vertical deposition is another method used to prepare RSF films that is typically achieved by dipping a clean glass surface into an RSF solution, and then drying it in an oven at 50 °C. This method, however, generates non-homogeneous structures, which show the presence of “valleys and ridges”. Recent studies have indicated that a poor cell attachment was achieved when using this deposition method of RSF films [104]. Temperature Controlled Water Vapor Annealing (TCWVA) is a physical method that can change the structure of RSF films to the insoluble Silk II state [97]. In this method, RSF films are casted into flat molds and placed in a constant temperature and humidity chamber at 65 °C with a relative humidity of 90% for 100 min. RSF films obtained via this method (Figure 4A) were successfully applied in skin TE applications, [97] which are described in more details below.

### 5.2. Mats and Artificial Fibers

Fiber spinning techniques, including electro-spinning, wet-spinning and dry-spinning, and are the most commonly used to make RSF mats or artificial silk fibers. The electro-spinning technique can be employed to make polymeric nanofibrous scaffolds, which can mimic properties of fibrous ECM components. RSF can be fabricated in a large scale and porous structure though electro-spinning, which is of great benefit for cell seeding in TE [105,106]. RSF mats, produced by electro-spinning, usually involve spinning solvents (e.g., polyethylene oxide (PEO)), which can adversely affect biocompatibility [39]. Jin et al. [107] reported RSF–PEO electro-spun mats were immersed in water for two days to remove the PEO solvent, and in return, the number of human marrow stromal (BMSC) cells attached on their surface increased. Additionally, electro-spinning allows for modified RSF mats to be produced by adding different moieties for extra functions. For example, the addition of cellulose ‘nanowhiskers’ (CNWs) [108] and polycaprolactone (PCL) [109] can strengthen the young’s modulus and tensile strength of RSF mats, whereas the addition of silver (Ag) [110] or titanium dioxide (TiO_2_) [111] nanoparticles confer enhanced antimicrobial properties to RSF mats. Recently, Yin et al. [98] developed a finite element model that expressed the mechanical response of RSF–PCL mats under biaxial tension. This model could be used to guide the design of RSF–PCL mats for TE applications. Wet-spinning also can be used to fabricate RSF fibers, but on the micrometer scale (fiber diameter) in contrast to nanofibers from electrospinning. Wet-spinning allows the tuning of fiber morphologies and properties, and allows the combination with other biomolecules whilst fabricating [36,112]. For example, Jacobsen et al. [99] reported RSF and fibronectin (Fn) silk fibers obtained from RSF solutions and fibronectin proteins via wet-spinning, which demonstrated better cell attachment to those made of pure silk fibers via wet-spinning. In contrast to the former methods, dry-spinning does not require the use of organic solvents or coagulation baths, which is environmentally friendly. In this context Zhang et al. [24] reported the fabrication of RSF–graphene oxide (GO) hybrid silk fibers obtained from aqueous RSF blends with graphene oxide via the dry-spinning technique. Compared with silk fibers, RSF–GO composite silk fibers showed good biocompatibility and enhanced mechanical properties that have great potential for TE applications. In addition, a newly developed approach uses centrifugal electrospinning (CES) and was shown to spin RSF nanofibers with better structural stabilities and thermostabilities than those obtained from electrospinning [113]. Moreover, compared to electrospinning, this method allowed for a higher production rate at lower cost and was able to quickly produce highly interconnected nanofiber nonwoven meshes [114,115].

### 5.3. Hydrogels

Hydrogels are water-swollen 3D polymer networks, which can be cross-linked via physical or chemical methods, and are excellent for the implementation of cell seeding and encapsulation in the development of tissue engineering applications [116]. To date, RSF hydrogels have been used with increasing popularity alongside other RSF morphologies, which is mirrored by the ever-increasing silk-based publication records [32]. Table 1 illustrates to date the developed fabrication techniques of RSF hydrogels.

Research shows that RSF hydrogel gelation kinetics can be modified from minutes to hours by adjusting pH, temperature, protein concentration, as well as the addition of precipitating agents. In general, during sol-gel transition of RSF solutions, the SF structural conformation changes from a random coil structure (Silk I) to a β-sheet conformation (Silk II) [117]. However, it is worthy to note that electro-gelation hold an exception to this, where the random coil conformation changes to α-helixes rather than β-sheet and the transition process is reversible by reversing the polarity of applied potential [118,119]. Cells can be encapsulated into RSF hydrogels that can be consequently used as a delivery system [120]. For example, Wang et al. [121] encapsulated human mesenchymal stem cells (HMSC) into sonication-induced RSF hydrogels, and reported proliferation and viability in static cultures after a week of in vitro cultivation. 

### 5.4. Sponges

Sponges are made up of interconnected porous structures that have been shown to closely mimic physiological environments in vivo [1]. RSF sponge scaffolds with different pores size can be formed by use of porogens, freeze-drying, and gas foaming fabrication techniques [73,136]. Sodium chloride (NaCl) particles are a classic example of a porogen and are added into SF solutions cast into Teflon (PTFE) molds. After scaffold formation, the salt is left to leach out of the construct (in di-water) [73]. This method leads to RSF sponge scaffolds with a highly homogeneous uniform pore size distribution, providing the NaCl particles added have a homogenous size distribution [136]. Another method of regulating the pore size of sponges is via freeze drying, here the freeze drying temperature, fibroin concentration, and pH of the RSF solution affect the pore size [137]. For example, Mandal et al. [138] reported that at fixed fibroin concentrations, the pore size decreased with decreasing temperature. In contrast, with a constant freeze-drying temperature but increasing fibroin concentration the pore size decreased. In addition, the pore size increased further with repeated freeze and thawing cycles [139]. Gas foaming techniques also can form RSF sponges. Ammonium bicarbonate added into fibroin solutions will sublimate in hot water aiding the formation of porous sponge structures [73]. Yan et al. [33] also combined the aforementioned and mixed NaCl particles in highly concentrated RSF solutions, followed by freeze drying, which showed a favorable stability in the formation of macro- and microporous structures. RSF sponges have been widely used in tissue engineering, especially in bone and cartilage, [1,26] because of excellent porosity and pore size control [140].

### 5.5. Micro-Patterning Structures

The Extracellular matrix (ECM) is made up of complex micro- and nano-scale topographies, which can affect cell behaviour. It is therefore important to try and mimic these topographies as much as possible, to ensure cell behaviour is similar in-vitro to in-vivo scenarios. Micro-patterning structures of RSF have been shown to affect the cell migration, proliferation, and adhesion [141,142]. At present, lithographic techniques are the most commonly used methods in micropatterning RSF biomaterials. These methods include, ultraviolet lithography (UVL) [143], soft lithography (SL) [144], electron-beam lithography (EBL) [145] and scanning probe lithography (SPL) [146].

**Figure 5:**
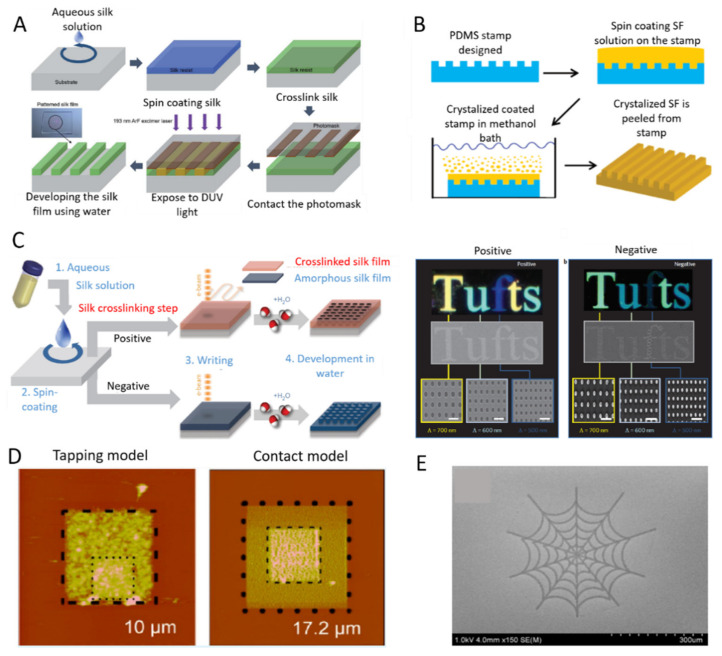
Micro-patterning of silk-based biomaterials. (**A**) Schematic diagram of ultraviolet lithography process which can form high-resolution silk fibroin micro-patterns by ArF (argon fluoride) excimer laser. (**B**) Schematic diagram of soft lithography of fabricating patterned silk films. (**C**) Schematic diagram of water-based electron-bean patterning on a silk film. Dark-field and electron microscopy images of silk nanostructures generated on positive and negative resist. (**D**) Atomic force microscopy (AFM) images of patterned silk films fabricated by AFM patterning in tapping mode and contact mode. (**E**) SEM images of micro-spider web fabricated by inkjet printing. Reprinted with permissions from [101,143,144,145,146].

UVL, as schematically shown in Figure 5A, is carried out by spin coating RSF onto silica substrates as a positive-tone photoresist, which was illuminated by argon fluoride excimer laser through a patterned chrome mask. After washing the exposed area with Di-water, the patterned RSF film showed diffracted colours with minimum line widths of 1 μm. It is important to note that, this process is water-based and does not require photoinitiators [143]. In contrast to UVL, SL is cheaper and requires fewer steps [147]. For example, Gupta et al. [144] spin-coated RSF onto polydimethylsiloxane (PDMS) stamps, and submerged them into a methanol solution. The crystallized RSF films were then peeled from the stamp, as shown in Figure 5B. In the case of EBL, shown in Figure 5C, RSF functioned as a resist material, the solubility of which could be regulated by different dosages of electron radiation. Therefore, amorphous RSF can be crosslinked while crystalline RSF can be de-crosslinked through electron bombardment. The RSF that has not been crosslinked can then be simply washed away with water. For example, RSF was spin-coated onto substrates to form RSF films. Then, for positive resist fabrication, inelastic collision of electrons with RSF resulted in protein degraded (de-crosslinked) into a water-soluble state, followed by washing away during the ‘water development’ process. In contrast, for negative resist fabrication, high doses electron beam bombarding on a water-soluble RSF solution resulted in crosslinking of the RSF into the water-insoluble state. After that, the ‘water development’ process washed away the area that has not been crosslinked, leaving the area exposed to the electron beam [145]. The reported critical feature sizes of UVL, SL, and EBL techniques are around 1.5 μm, 40 nm, and 20 nm, respectively [148]. Another technique namely SPL, as shown in Figure 5D, also offers high precision and resolution by means of an atomic force microscopy (AFM) tip. One type of SPL uses AFM as a tool to pattern RSF films under aqueous environments via tapping mode or contact mode [146]. Piezoelectric-based inkjet printing can be used in large-scale fabrication where no cast or spin coating is needed for inducing structural transformation of SF [148]. Inkjet printing can print functional inks in computer-aided design (CAD) patterns, such as RSF inks mixed with enzymes, [34] growth factors, gold nanoparticles, antibiotics, or other moieties on different surfaces suitable for tissue engineering applications. Tao et al. [101] reported inkjet printing of a spider web (Figure 5E) pattern using RSF as the ink and the thickness of the pattern could be regulated by controlling the amounts of printed drops.

### 5.6. 3D Bioprinting Structures

**Figure 6:**
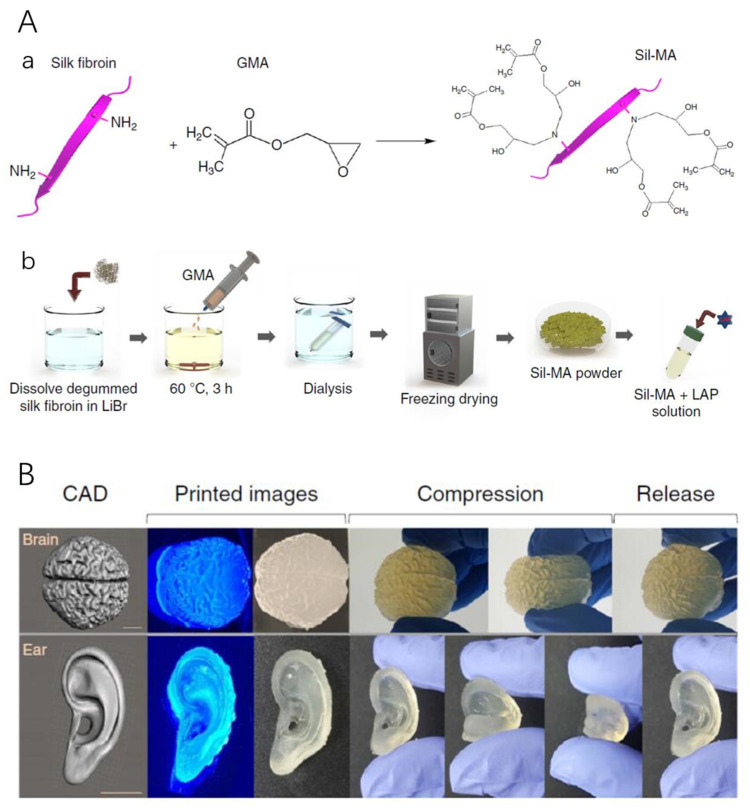
(**A**) Regenerated silk fibroin (RSF) was chemically modified with glycidyl methacrylate (GMA) to form (Sil-MA) as a pre-hydrogel. (**a**) RSF covalently immobilized with GMA, generating a vinyl double bond as a UV-crosslinking site. (**b**) Schematic diagram of the methacrylation process of SF; LAP represents Lithium phenyl(2,4,6-trimethylbenzoyl) phosphinate which is a photoinitiator. (**B**) Representative 3D printed models (brain and ear) via a digital light processing (DLP) printer using Sil-MA as a bioink, showing complex structure reflecting their CAD images. Reprinted with permission from [154].

Sponges prepared with typical methods have no defined internal pore architecture which can obstruct cellular response. Bioprinting is a bottom-up additive manufacturing technology that can be used to manufacture complex structures via CAD design at high definition. For example, biocompatible hydrogels can be printed via 3D extrusion bioprinting. It is possible to encapsulate cells in hydrogels giving them mechanical support in a 3D environment similar to their native tissue [149]. Although 3D bioprinting has been applied in tissue engineering, there are still many challenges to overcome, including a limited range of materials and choice of cell types [150]. RSF is a unique material for 3D printing owing to its biocompatibility and polymorphic nature [21]. RSF can be printed via inkjet printing to fabricate “nest” shapes. RSF printed “nests” of 70–100 µm diameters, were stabilized by ionic pairing, followed by a drying process to form silk II crystalline secondary structures, and could act as anchored nests for cell incubation and proliferation [151]. Das et al. [152] reported 3D bioprinting RSF-gelatin scaffolds which could be used in culturing human nasal inferior turbinate tissue-derived mesenchymal progenitor cells. The sonication treated RSF-gelatin hydrogels possessed higher β-sheet content compared to that of tyrosinase enzyme-treated hydrogels, further to this only the sonication produced RSF-gelatin hydrogels demonstrated enhanced osteogenic differentiation. In addition, a recent study by Rodriguez et al. [102] reported the successful printing of RSF, synthetic nanoclay (Laponite), and polyethylene glycol (PEG) scaffolds via extrusion-based 3D Printing. Here, a key advantage is that gelation of the scaffolds occurs during the printing process and therefore there is no need for additional post-processing, such as chemical or photochemical crosslinking. This allowed for simple and rapid fabrication of complex geometries of the biomaterials down to the microscale. Generally, 3D printed RSF scaffolds are macroscopic in structure, but can be regulated into mesostructures and nanostructures by using mechanical stresses and dopants. For example, Sommer et al. [153] reported the pore size of an RSF structure could be regulated by adding sacrificial monodisperse organic microparticles with varying sizes into RSF-based inks to create well-defined porous RSF scaffold structures. Recently, Kim et al. [154] reported RSF can be chemically modified with glycidyl methacrylate (GMA) to form a printable bioink (Sil-MA) (Figure 6A) which could be printed to form complex structures, e.g., brain and ear, via a digital light processing (DLP) 3D printer (Figure 6B). The produced 3D scaffolds possessed strong mechanical properties, which can be used in cartilage TE applications. Following this work, Ajiteru et al. [155,156] further improved the properties of the Sil-MA bioink by conjugating it with reduced graphene oxide (rGO) to form a composite bioink, which was shown to exhibit better thermal stability, as well as higher solubility.

## 6. Application of Silk Fibroin in Tissue Engineering

### 6.1. Bone Tissue Regeneration

Bone is a specialized connective tissue, and is composed of 35% organic parts and 60% inorganic matrix. More than 90% of the organic extracellular matrix of bone is made up of collagen and the rest contains hyaluronan, proteoglycans, bone sialoprotein, osteopontin, osteonectin, and osteocalcin [157,158]. Hydroxyapatite (HA) is the major component of the inorganic mineral phase of bone, while the remaining is composed of inorganic salts and carbonate [159]. This means that collagen and HA are the major components of bone tissue, which enhances the strength and hierarchical architecture of bone [160]. Designed scaffold materials for the use in bone tissue engineering should guarantee matrix toughness and allow for ECM deposition. SF has high toughness, mechanical strength and proven biocompatibility which has already been widely studied in bone TE [161]. For example, RSF scaffolds have been shown to promote osteogenic differentiation of human mesenchymal stem cells (HMSC) in vitro. These constructs have been shown to heal femoral defects in vivo in nude rat models [162]. Meinel et al. [140] demonstrated that after initial incubation in bioreactors for 5 weeks, porous SF-based scaffolds could be implanted into cranial defects in mice and showed advanced bone formation within 5 weeks, in vivo.

RSF scaffolds are used in combination with other biomaterials such as collagen, or calcium phosphate-based inorganic components to enhance osteogenic properties [28,163]. For example, HA–RSF porous scaffolds were fabricated through an alternate soaking process in CaCl_2_ andNa_2_HPO_4_, or alternatively by mixing NaCl particles with HA and then mixing these with RSF solutions [164,165]. These composite scaffolds were shown to have better osteoconductivity and exhibited an enhanced formation of tissue engineered bone, compared to unmodified RSF scaffolds.

Bone morphogenetic protein (BMP)-2 and BMP-7 are FDA recognised growth factors that can support bone formation and regeneration [166]. It has been shown that RSF combined with these growth factors together with HMSCs exhibited enhanced osteoblast adhesion and differentiation, stimulated alkaline phosphatase activity and promoted bone formation in vivo [167,168]. In addition, Li et al. [169] reported the modification of electrospun RSF mats with BMP-2 and HA nanoparticles, which support HSMCs differentiation and growth and resulted in more calcium deposition in comparison to RSF mats only. Moreover, demineralized bone matrix (DBM) powder or particles are mainly composed of collagen and BMP which also has osteoinductive and osteoconductive. Ding et al. [170] reported RSF as a carrier for loading DBM. This carrier can form stable porous structures and has been shown to promote osteogenesis in mice together with bone marrow stem cells (BMSC).

Rapid and thorough vascularization is required in order to increase the success of bone regeneration. For example, RSF matrices pre-incubated with osteoblasts in vitro and then implanted into mice showed enhanced vascularization in vivo [171]. In addition, co-cultures of endothelial cells and osteoblasts in RSF scaffolds in vitro showed the formation of microcapillary-like structures and pre-vascular structures [172,173]. Subsequently, pre-formed microcapillary-like structures implanted into immune-deficient mice, not only survived, but successfully interfaced with the host vasculature, and further stimulated the host capillaries for vascularization [174]. Further to this the vascular endothelial growth factor (VEGF) could not only promote osteoblast differentiation but also caused neovascularization [175]. In this context, Farokhi et al. [176] embedded VEGF into RSF–calcium phosphate–poly(lactic-co-glycolic acid) scaffolds. The results indicated that the scaffolds maintained about 83% bioactivity after VEGF release up to 28 days in vitro. For in vivo study, the neo-bone formation in defects site of rabbits after implanted for 10 weeks. Another study conducted by Zhang et al. [120] reported that a sonicated silk hydrogel carrier loaded with BMP-2 and VEGF could promote both osteogenesis and angiogenesis in rabbit’s maxillary sinus floor after implanted for 12 weeks.

### 6.2. Cartilage Tissue Regeneration

Cartilage is avascular and aneural connective tissue surrounded by a dense ECM and lacks the innate ability to self-repair after injury degeneration. Collagen and proteoglycans make up the main parts of the cartilage ECM, which can provide adequate mechanical properties for tissues in vivo [177,178]. Therefore, maintaining and preserving this tissue is an important aspect of tissue engineering. SF scaffolds can be used to enhance the production of cartilaginous ECM [179] and owing to its tuneable properties the resulting scaffolds can be fabricated into different morphologies [180]. For example, porous RSF scaffolds combined with HMSCs can provide zonal structures similar to that of native cartilage tissue, which was shown by Wang et al. [180] after 3 weeks of incubation, HMSCs grew along the chondrogenic route within the scaffold [181]. In addition, the Insulin-like growth factor I (IGF-I) can promote different progenitor cell growths, which can be loaded into porous RSF scaffolds promoting chondrogenic differentiation of HMSCs [182].

**Figure 7:**
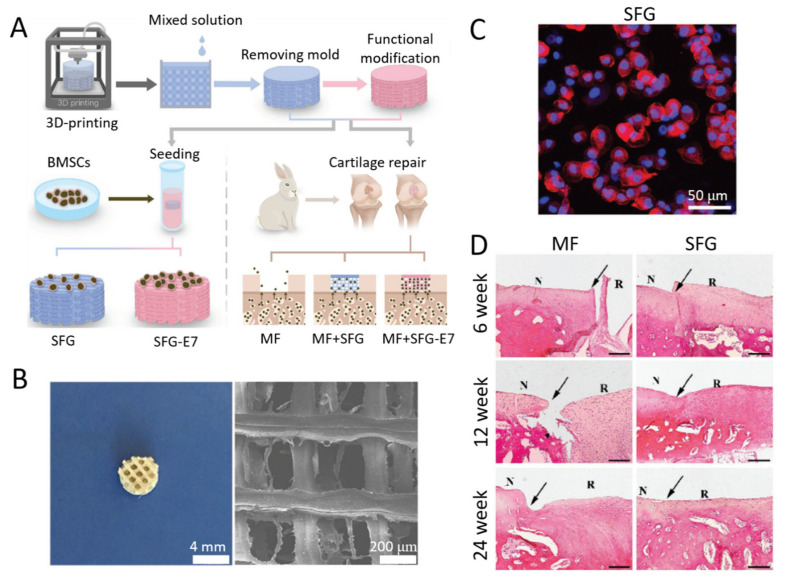
(**A**) Schematic diagram illustrating the fabrication of a 3D of scaffold made via Bioprinting up to the final in vivo implantation. (**B**) Microscopy and SEM images of the RSF-Gelatin scaffold (mixture of SF solution and gelatin solution at a mass ratio 1:2). (**C**) Phalloidin/Hoechst assay of chondrogenic morphology on the SFG scaffold after 21 days incubation. (**D**) Hematoxylin-eosin staining of repaired cartilage at 6, 12, and 24 weeks. (MF represents the microfracture control group; N represents normal cartilage; R represents repaired cartilage; the margins between repaired and normal cartilage are indicated by black arrows; scale bar: 200 μm). Reprinted with permission from [188].

Other natural biopolymers can be blended with RSF to produce biocompatible cartilage constructs. One example is chitosan, which can provide sufficient support to chondrocytes due to the existing glycosaminoglycan residues [183]. Both Bhardwaj et al. [184] and Silva et al. [185] investigated this, showing that chitosan could increase cell attachment, proliferation, and chondrogenic phenotype of chondrocytes or chondrocyte-like cells. Another biopolymer combination that has been studied is RSF-Gelatin. Gelatin is a partial derivative of collagen, and both collagen and gelatin possess the ability to promote chondrogenic differentiation [186]. RSF–collagen dense mats fabricated by electrospinning and seeded with MSCs showed better chondrogenic differentiation of MSCs and promoted expression of cartilaginous matrix compared to collagen-only dense mats [187]. It is assumed this might be caused by the increase in scaffold strength. In addition, Wang et al. [30] fabricated porous RSF–collagen scaffolds combined with poly-lactic-co-glycolic acid (PLGA) microspheres which exhibited good cell affinity and promoted articular cartilage in rabbits. Recently, Shi et al. [188] reported that a mixture of SF solution (6.9% *w/v*) and gelatin solution (6.9% *w/v*) at a mass ratio 1:2 could be used to fabricate RSF–Gelatin scaffolds with good degradation and mechanical properties via 3D printing for the use in cartilage repair (Figure 7A,B). SFG scaffolds have chondrogenic differentiation abilities of bone marrow stem cells (BMSC), the native round shape of chondrogenic cells could be observed after 21 days of in vitro incubation (Figure 7C). In addition, it has been shown that SFG scaffolds implanted into defective rabbit cartilage positions repaired the cartilage defect after 24 weeks (Figure 7D). Except for the above mentioned, RSF also can be blended with other biopolymers such as cellulose, hyaluronic acid, agarose, and poly(D,L- lactic acid) to provide sufficient support to chondrogenesis [189,190,191,192].

It has been shown that, the mechanical and structural characteristics of RSF-based scaffolds can be improved by argon plasma treatment [193,194,195]. For example, Baek et al. [193] reported that porous RSF scaffolds treated with microwave-induced argon plasma exhibited a significantly increased hydrophilicity and therefore increased chondrocyte adherence, and proliferation. It has been shown that cells seeded on the RSF scaffolds and then incubated in physically stimulated bioreactors under physiological conditions could further improve cartilaginous constructs [196,197]. For example, the amounts of glycosaminoglycan, total collagen, collagen II, and DNA along with cartilage-related gene produced by cells increased significantly by seeding porous SF scaffold with HMSCs and incubated in perfusion bioreactors for 4 weeks. Additionally, the same study found that the mechanical stiffness of the stimulated scaffold also increased in comparison to a static culture [196]. These results indicate that hydrodynamic factors, as well as cell types [198], scaffold architectures, e.g., pore size and distribution are key components to successful constructs for cartilage TE [36].

### 6.3. Ligament and Tendon Tissue Regeneration

Ligament and tendon tissues are composed of collagen and fibrocytes which are made up of a dense fibrous connective tissue, which can be easily impaired, and severely lacks the ability of natural regeneration [199,200]. Due to its unique mechanical properties (such as high toughness values and good elasticity) and structural integrity, RSF scaffolds have become a preferred biopolymer for the use in ligament and tendon TE [36]. In 2002, the first RSF matrix was successfully implemented in engineering an anterior cruciate ligament (ACL) that matched the mechanical properties of the human ACL [72]. Based on these promising results, researchers started fabricating knitted SF-based scaffolds for the regeneration of ligaments and tendons. For example, Liu et al. fabricated web-like SF sponges on knitted scaffolds on which HMSCs were seeded, these scaffolds are more cellular actively compared to RSF hydrogel knitted scaffolds. The results demonstrated SF-based knitted scaffolds proved structural strength, while the web-like microporous RSF sponges can enhance cellular activity [201]. Subsequently, Fan et al. implanted RSF porous knitted scaffolds with MSCs into rabbit [202] and pig [203] models to regenerate ACL. After 24 weeks of implantation, the direct ligament–bone insertion with four zones (bone, fibrocartilage, mineralized fibrocartilage, and ligament) in rabbit and three zones (bone, Sharpey’s fibers, and ligament) in pig was reconstructed, which was similar to native structures of ACL-bone insertion. The tensile strength of regenerated ligaments also compared to the mechanical properties of the native ligaments. In addition, Chen et al. [204] combined RSF knitted scaffolds with collagen and then implanted these into rabbit medial collateral ligament (MCL) defected regions, which was shown to promote scaffold–ligament interface healing, compared with untreated MCL or only SF knitted scaffold.

An arginine-glycine-aspartic acid (RGD) peptide sequence can also be immobilized onto RSF scaffolds and has been shown to promote the attachment of BMSC cells leading to higher human bone marrow cells and ligament fibroblast formation [205]. Additionally, sequential administration of growth factors, including epidermal growth factors, transforming growth factor-β and basic fibroblast growth factor, induced BMSCs cells to proliferate and differentiate on RGD-coupled RSF scaffolds, which can boost the development of ligament tissue. Growth factors stimulate biochemical and mechanical properties, thereby inducing cell differentiation toward a fibroblast lineage and enhanced matrix in-growth, as well as collagen production [206,207,208].

In conclusion, blending RSF with natural biomaterials such as collagen type I, hyaluronic acid and gelatin and synthetic materials, e.g., polyelectrolyte and PLGA leads to the enhancement of scaffolds for use in the reconstruction of ligament and tendon connective tissues [209,210,211,212,213].

### 6.4. Skin and Wound Tissue Regeneration

**Figure 8:**
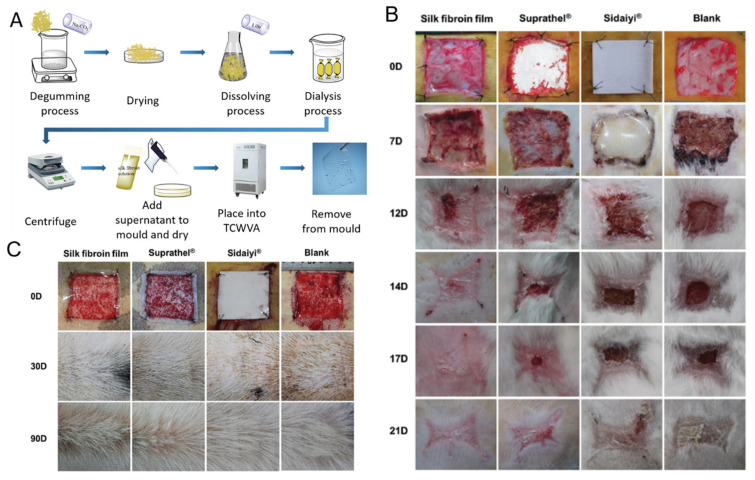
(**A**) Schematic diagram of silk fibroin films via Temperature Controlled Water Vapor annealing (TCWVA). (**B**) RSF films implanted into full-thickness skin defects in rabbit models compared to Suprathel, Sidaiyi, and untreated tissue at 0, 7, 12, 14, 17, and 21 days. (**C**) RSF films implanted into full-thickness skin defects in a porcine model and compared to Suprathel, Sidaiyi, or untreated at 0, 30, and 90 days. Reprinted with permission from [97].

The skin holds a critical role as the first line of defense to infectious organisms. Epidermis and dermis are the main layers of skin, which mainly consist of keratinocytes and ECM (mainly collagen and elastin). In cases with extreme loss of skin integrity, e.g., severe burns, this can lead to disability and even death [214,215]. RSF biomaterials have been shown to influence the attachment of keratinocytes and fibroblasts [106] and are widely applied to skin regeneration in TE. Recently, Zhang et al. [97] reported RSF films could be implanted into full-thickness skin defects in rabbit models (Figure 8A,B) and porcine models (Figure 8C), which significantly reduced the healing time and showed better skin regeneration compared to current commercial wound dressings. In clinical trials, RSF films have also been shown to significantly reduce the healing time and lower the probability of adverse events, compared to commercial wound dressings. Additionally, RSF mats coated with antibacterial silver nanoparticles (AgNPs) could be used as antimicrobial wound dressings to inhibit the growth of Staphylococcus aureus and Pseudomonas aeruginosa [110]. The hydrophilicity of RSF nanofibers has been shown to increase after O_2_plasma treatment, which has been shown to promote human keratinocytes and fibroblasts activities [216].

Appendages on the skin (e.g., hormonal glands and hair) make skin tissue complex [36]. Chitosan has been widely used in skin TE, due to its biocompatibility, biodegradability and antimicrobial ability, as well as being known to promote collagen formation from fibroblast cells, which increases the tensile strength of the regenerated tissue in the defected area [215]. Cai et al. [29] fabricated RSF–chitosan scaffolds via electrospinning and found the mechanical strength increased with increasing RSF concentration, as well as an increase in antimicrobial activity with increasing chitosan concentration. Moreover, RSF–chitosan scaffolds were shown to promote cellular proliferation and antimicrobial property against *Escherichia coli* [29,217]. Furthermore, alginate dialdehyde (ADA) enhances cell proliferation and attachment and has a lower toxicity when used as a crosslinker [218]. Therefore, ADA can be used to crosslink RSF–chitosan scaffolds in defected skin areas and shows good water absorption, high water transmission and increased cell activity [219]. Guang et al. [220] reported chitosan coatings on porous RSF scaffolds via a hydrogen-bonding technique to form 3D RSF–chitosan scaffolds, and then implanted these into a rat wound. Their data showed the wound was fully repaired after 21 days and without any teratogenic effects and infections. In comparison non-mulberry RSF from *Antheraea assama* (*A. assama*) was also shown to be a promising material for skin TE. This is because it naturally contains the RGD peptide sequence that promotes cell attachment [221]. Chouhan et al. [222] fabricated RSF hydrogels by blending SF solutions isolated from *A. assama* and *B. mori*, which promoted the differentiation of primary human dermal fibroblast and keratinocytes cells in vitro. In addition, the blended SF solutions were injected into third-degree burn wounds in-vivo and formed gels that firmly adhered to the wounds. The blended RSF hydrogel not only acted as a supportive matrix for skin repair, but also showed transition stages from inflammation to proliferation.

The above mentioned illustrate the vast capabilities the RSF scaffold materials and how they can be blended with natural and synthetic materials, such as dextrose, 2,2,6,6-Tetramethyl-1-piperidinyloxy (TEMPO)-oxidized cellulose nanofiber, Manuka honey, Ag particles, collagen, chitin to increase mechanical properties, decease wound infections, and improve wound healing [215,223,224,225,226,227,228]. Therefore, the use of composite RSF scaffolds were able to provide overall better results to pure RSF scaffolds in skin TE applications.

### 6.5. Tympanic Membrane Tissue Regeneration

Tympanic membrane (TM) is a transparent structure located between outer and middle ear, whose functions are receiving sound vibrations and protecting the middle ear. TM is composed of the epidermal outer layer, fibrous middle layer and mucosal inner layer, which mainly consist of keratinocytes, fibroblasts, and collagen (type II and type III). TM perforations are normally caused by middle ear infections or traumatic ruptures caused by mechanical trauma and pressure blasts. If the rupture has not self-repaired within 3 months, it will become a chronic perforation, which can lead to hearing loss and recurrent infections [229,230,231]. The excellent properties of RSF as mentioned above make it an ideal material for tympanic membrane TE, supporting the growth and spread of keratinocytes derived from human TM cells. Shen et al. implanted RSF films into rat and guinea pig models to regenerate acute [232,233,234,235,236]. TM perforations using onlay myringoplasty. After implantation, a perforation closure for both rat and guinea pig models were observed after 7 days, where no recovery was observed in the control groups. In addition, RSF films were shown to not only repair TM perforation but also accelerate the regeneration of TM, leading to a significantly faster hearing recovery. Furthermore, Shen et al. [237] demonstrated RSF films showed no significant macrophage response in host tissue, less inflammation, and was degradable in vivo. In addition, Allardyce et al. [238] reported that RSF membranes possess good acoustic energy transfer capability and excellent tensile strengths to cartilage, indicating the great potential of these membranes to regenerate chronic TM perforations in vivo.

## 7. Conclusions

The future of modern tissue engineering is to regenerate and replace damaged tissue and organs. This means that implanted scaffolds should be fully integrated into the surrounding tissue without any immune response or adverse effects. Silk Fibroin extracted from silkworm cocoons, is an FDA approved biomaterial and has been widely recognized for use in TE applications, due to its unique biomedical properties, mechanical performance and tuneability. SF has been fabricated into various morphologies including films, mats, artificial fibers, sponges, and hydrogels, which have all been successfully deployed in a large variety of TE applications. Recent advances have played particular emphasis on bio-nanotechnology technologies, such as micropatterning and 3D bioprinting to fabricate SF multi-level structures with high structural definition down to the nanoscale. This has been shown to be advantageous for cell proliferation, differentiation, migration, and adhesion in many studies. Overall, silk is a versatile biomaterial which has shown promising applications in TE.

## 8. Prospects and Challenges

As was reported by Thakur et al., 2D nanomaterials possessing high aspect ratios and ultrathin structures could interact with the polymers to enhance their mechanical properties [239]. Additionally, some specific pattered 2D nanomaterials could have similar effects to growth factors on the enhancement of cell differentiation [240]. SF has shown great potential together with 2D nanomaterials to increase its mechanical properties for future bone, cartilage ligament and tendon tissue engineering applications. In skin and wound applications, the production of SF matrices built on particular morphologies has demonstrated promise in decreasing the risk of scar tissue in patients. However, at present, their clinical applications are still scarce and therefore more research needs to be conducted to move towards clinical trials and FDA approved products based on this excellent biomaterial.

In addition, pure RSF has been shown to exhibit poor attachment and proliferation for some cells, such as neuronal cells [27]. Therefore, research suggests that SF should be used in conjunction with other moieties, e.g., ECM or synthesized peptides to improve its functionality to enhance its applicability in other fields of TE. On the other hand, four-dimensional (4D) printing (when 3D printing combined with ‘Time’) has emerged and became an emerging technology and attractive topic, which can overcome some limitations of 3D printing, such as the creation of the sophisticated dynamics of native tissues [241] and optimize the functional responses of cell-constructs interactions [242]. ‘Time’ is defined as printed 3D biocompatible scaffolds that continue to evolve over time while they are printed [243]. The materials chosen for 4D printing should possess biocompatibility and reshape or change their function by means of external stimuli including temperature, water, magnetic fields, osmotic pressure, and light [243]. Very recently, Kim et al. [244] described a 4D printing system based on Sil-MA hydrogels and DLP, which has been successfully applied in the regeneration of damaged trachea of rabbits. Therefore, RSF could be a key biomaterial that can be used in bioink formulations, illustrating its great potential in future 4D bioprinting.

## Figures and Tables

**Table 1:** Key silk fibroin hydrogel fabrication techniques.

Methods	Fabrication Techniques	Comments
Chemically induced gelation	Salts	Salts can promote protein-protein association for example the addition of Ca^2+^ ions reduce the gelation time of RSF solution [122,123].
	Polymer agents	Polymer agents, such as polyethylene glycols and PEO, have been shown to promote protein-protein associations, and protein aggregation through volume exclusion and movement of water by osmosis [122,123].
	Organic solvents	Alcohols are the most common used among organic solvents, which can induce structural conformation changes of RSF from α-helix to β-sheet structures [124].
	Surface active agents	Surface active agents readily bind with proteins leading to protein unfolding and aggregation [125]. For example, adding the anionic surfactant sodium dodecyl sulfate (SDS) into RSF solutions and incubating at 60 °C can induce stable hydrogels with good mechanical properties [100].
	Small neutral additives	Small neutral additives through their ionic strength and/or specific interactions with proteins can influence protein aggregation.125 For example, the addition of glycerol (30%; *v/v*) can reduce the gelation time of RSF solution and has been applied in biomedical applications [126,127].
	pH	As the pH of RSF solution is adjusted near the isoelectric point (PI = 3.8–3.9), stable hydrogels can be formed as well as reduced gelation time of RSF scaffolds [128]. This is because the pH of protein solution near its isoelectric point can induce protein precipitation [32].
	High pressure CO_2_	High-pressure CO_2_ as a volatile acid can be used as a fine tuning adjustment of the solutions pH, therefore, RSF solutions subjected to high-pressure CO_2_ at 60 bar, has been shown to form stable hydrogels within 2 h [129].
	Chemical crosslinking	Chemical crosslinking agents (e.g., hydrogen peroxide and horseradish peroxidase) can be used to covalently crosslink phenol groups of tyrosine residues on silk fibroin proteins to form highly elastic RSF hydrogels [130].
	Chemical coupling	Diazonium coupling chemistry can functionalize tyrosine residues of SF protein, resulting in an adjustment of the hydrophobic and hydrophilic properties, giving rise to the ability to rapidly produce controlled RSF hydrogels from as little as 5 min to two hours [131].
Physically induced gelation	Temperature	The gelation time of RSF solutions decreases with increasing temperature, this is because molecular collisions increase with respect to temperature [117,123].
	Shear force	A strong enough shear force applied to an RSF solution can promote molecule-molecule interactions and improve concentration fluctuation, resulting in gelation and aggregation phenomena [132,133]. Vortex mixing is the way to initiate RSF gelation due to the high shear forces applied to the solution [134].
	Ultrasound	Sonication can lead to local areas of extreme pressure and temperature, resulting in gelation and aggregation [135].
	Electric fields	Applying electric fields across RSF solutions leads to local pH decreases and thus silk protein aggregates [134].
